# Prognostic Value of Colorectal Cancer Biomarkers

**DOI:** 10.3390/cancers3022080

**Published:** 2011-04-19

**Authors:** Paolo Bianchi, Luigi Laghi, Gabriele Delconte, Alberto Malesci

**Affiliations:** 1 Laboratory of Molecular Gastroenterology, IRCCS Istituto Clinico Humanitas, Rozzano, Milano 20089, Italy; E-Mail: paolo.bianchi@humanitasresearch.it (P.B.); 2 Department of Gastroenterology, IRCCS Istituto Clinico Humanitas, Rozzano, Milano 20089, Italy; E-Mails: luigi.laghi@humanitas.it (L.L.); gabriele.delconte@humanitas.it (G.D.); 3 Department of Translational Medicine, University of Milan, Milano 20089, Italy

**Keywords:** colorectal cancer, prognostic biomarkers, predictive biomarkers

## Abstract

Despite the large amount of data in cancer biology and many studies into the likely survival of colorectal cancer (CRC) patients, knowledge regarding the issue of CRC prognostic biomarkers remains poor. The Tumor-Node-Metastasis (TNM) staging system continues to be the most powerful and reliable predictor of the clinical outcome of CRC patients. The exponential increase of knowledge in the field of molecular genetics has lead to the identification of specific alterations involved in the malignant progression. Many of these genetic alterations were proposed as biomarkers which could be used in clinical practice to estimate CRC prognosis. Recently there has been an explosive increase in the number of putative biomarkers able to predict the response to specific adjuvant treatment. In this review we explore and summarize data concerning prognostic and predictive biomarkers and we attempt to shed light on recent research that could lead to the emergence of new biomarkers in CRC.

## 1. Introduction

In clinical practice, the main aim of a staging system is to provide the most accurate prediction of the tumor's clinical and biologic behavior. Colorectal cancer (CRC) is a major cause of cancer mortality worldwide, accounting for approximately 500,000 deaths per year [1]. A significant proportion of patients presenting with stage I, II, or III disease (75%) can be treated with surgery alone or in combination with chemotherapy, and have a 5-year survival rate of 93.2%, 82.5%, and 59.5%, respectively, compared with only 8.1% survival rate of patients harboring stage IV disease [2]. The probability of distant metastasis and the response to chemotherapy are the most important clinical variables that directly affect patient outcome. Even if surgery remains the primary treatment for CRC, adjuvant chemotherapy is routinely employed to treat those patients at high risk of developing recurrence or those who already have metastatic disease at the time of diagnosis.

Despite the extensive use of chemotherapy, mechanisms involved in clinical response remain elusive. What we do know is that a significant proportion of patients receiving chemotherapy do not derive any advantage. It becomes essential to identify subgroups of patients who may benefit from adjuvant therapy to avoid a potentially toxic over treatment and an unprofitable financial burden for the health care system [3,4]. Therefore, to understand the reasons for treatment failure and hence develop methods able to predict which patient group is at risk of disease relapse and which group may benefit from a specific medical treatment, are the primary goals in the clinical management of patients with CRC.

Currently, the gold standard for predicting the outcome of CRC patients remains the AJCC/UICC (American Joint Committee on Cancer and Union for s concerning the effect of a specific therapeutic intervention. Clearly, this distinction should be considered with an open mind, as the border between natural history and treated disease is rapidly disappearing, leaving room for a multiplicity of pharmacological agents which are often sequentially used during disease course. Currently, the TNM staging system is the only valid prognostic marker in predicting the outcome of CRC patients [6-11], while gene mutations are gaining their place as predictive biomarker in a clinical scenario (see *KRAS* mutations below).

Several proteins and genetic markers have been described in an attempt to improve prognostic information and to predict the benefit from systemic treatment. Unlike other types of cancer, with the exception of *KRAS* mutation, none of the studied markers has entered into the clinical management of colorectal cancer so far [12]. In breast cancer, for example, hormone receptor status is linked to patient outcome. In breast cancer again, a cancer gene expression profile that identifies a worse prognosis [13,14] has been approved by the FDA (US Food and Drug Administration) as a method to support clinical decision making, and it is now clear which patients can benefit from treatment with trastuzumab based on epidermal growth factor receptor (EGFR) status. Thus, translational research failed to validate most of the putative CRC biomarkers, resembling the general scenario in clinical oncology [15]. This is largely due to discrepancies arising between initial research reports and subsequent validation studies. Inconsistencies are usually ascribed to methodological issues, including study design, employed assays, and statistical analysis. Accordingly, recommendations for REporting tumor MARKer prognostic studies (REMARK) have been suggested by the NCI-EORTC (National Cancer Institute— European Organization For Research And Treatment Of Cancer) working group on cancer diagnostics [15]. These guidelines were aimed to improve the general quality of reports on tumor biomarkers, and thus the interpretation of their usefulness. In this article we review the current status of prognostic and predictive biomarkers in CRC, evaluate their clinical usefulness, and explore future perspectives.

## 2. Prognostic and Predictive Biomarkers in Colorectal Cancer

### 2.1. Genetic Phenotypes of CRC

Our knowledge of the molecular pathogenesis of CRC has facilitated the identification of a number of promising biomarkers. Currently, most of the studied prognostic markers are somatic mutations acquired by cancer cells, for which biological rationales support activity on disease progression. Most of these biomarkers play an important role in the adenoma-carcinoma sequence proposed by Vogelstein [16]. The latter is a simplified model of stepwise accumulation of mutations in key genes or specific genetic loci: Disruption of WNT signaling pathway, reduced or loss of expression of SMAD4, activation by point mutation of *K-RAS* and *BRAF* protoncogenes, loss of heterozygosity (LOH) on chromosome 18, and LOH plus inactivating mutations of *TP53* tumor suppressor gene.

Colorectal tumors represent a heterogeneous disease with respect to the molecular alterations that they accumulate, although these are categorizable according to two main specific phenotypes of genomic instability. These instability phenotypes are considered as alternative mechanisms driving of colorectal tumorigenesis. The most common is chromosomal instability (CIN) affecting about 85-90% of CRCs. It is characterized by the presence of large structural or numerical alterations of the chromosomes in cancer cells [16-19]. The CIN phenotype seems to originate from molecular alterations in several cellular processes, including aberrant expression or mutations in key genes of the mitotic checkpoint, defects in the formation of mitotic spindle microtubules, and telomere dysfunction, yet its precise mechanism remains elusive [20]. The CIN phenotype has originally inspired the model of accumulation of mutations recapitulated by the adenoma-carcinoma sequence [16].

The remaining 10–15% of CRCs is characterized by a specific molecular phenotype displaying insertion and deletions in repetitive DNA tracts (microsatellite) and hence referred to as microsatellite instability (MSI) [21,22]. Microsatellites are short repetitive DNA nucleotide sequences prone to frameshift mutations and base-pair substitutions during replication. If the mismatch repair system is malfunctioning, these mutations within microsatellite sequences result in genomic instability [23-25]. The underlying mechanism of this phenotype is the loss of function of DNA mismatch repair genes (MMR) involved in maintaining the integrity of post-mitotic DNA. These MMR genes include *hMLH1, hMSH2, hMSH6*, and *PMS2*. About the 7–10% of sporadic MSI CRCs are characterized by the loss of expression of hMLH1 by promoter hypermethylation [26-28]. This phenotype is also present in about 3% of CRCs occurring in patients with a germline mutation in one of the MMR genes *(hMLH1, hMSH2, hMSH6*, and *PMS2)*, having hereditary nonpolyposis colorectal cancer (HNPCC or Lynch syndrome) [29-33]. Cancer cells with MSI exhibit a stable kariotype and frameshift mutations in specific genes (called *target genes)* that harbor microsatellite-type sequences (chiefly, *TGFβRII, CASP-5, BAX, hMSH6, hMSH3, AXIN2, TCF4, MBD4)*. Cancers with MSI tend to have more proximal location, poor differentiation, mucinous histology and high lymphocytic infiltration [34-41].

### 2.2. Genetic Phenotypes as Prognostic Markers

In the last two decades, basic science studies have initially identified CIN and MSI, and translational studies followed by meta-analyses [42,43] later proved the prognostic advantage of MSI CRCs. Clinical studies demonstrated that this advantage is mainly due to the minor metastatic potential of MSI CRCs, as displayed by a low frequency of stage (III and) IV MSI CRCs [39,44].

In contrast, the predictive value of MSI is still controversial. Elsaleh *et al.* showed that MSI was a factor predictive of response to the 5-FU-based adjuvant therapy in stage III MSI CRCs [45]. In contrast to this result, several studies have shown that MSI CRC patients do not benefit from 5-FU based therapy, as compared to patients with microsatellite stable (MSS) cancer [42,46-48]. On the basis of these results, MSI status should be taken into consideration, especially in stage II CRC patients, to address decision making concerning the use of adjuvant therapy [49]. MSI can be considered a strong and well validated prognostic marker and in an appropriate clinical setting, such as stage II patients, can be used in the decision making process, as the favorable outcome of patients with MSI CRC suggests that they could be spared adjuvant chemotherapy [50]. However, the clinician should consider that the role of MSI as prognostic and predictive marker in the adjuvant setting may be influenced by mutations of other key genes involved in colorectal carcinogenesis, such the BRAF gene [51].

### 2.3. “Epigenomic Instability”

It has also been suggested, mirroring the concept of genome-wide instability underlying cancer growth, that CRC can also display a separate type of instability, that is, “epigenomic instability”. Epigenomic instability comprises either global hypomethylation or a molecular phenotype named the “CpG island methylator phenotype” (or CIMP). The CIMP was operatively defined as hypermethylation at three or more marker loci, resounding MSI definition [52]. CIMP was mainly associated with MSI phenotype in sporadic MSI patients who do not harbor any germline mutation in the MMR genes that defines HNPCC [52]. Patients showing this phenotype are defined as having CIMP-high (CIMP-H) [53,54] or CIMP-1 [55]. Tumors with a hypermethylated phenotype, (with or without *KRAS* mutation), and a low level of methylation were classified as CIMP-low [56] or CIMP-2 [55]. Heavy methylation is strongly associated with the *BRAF^V600E^* mutation, which identifies almost two third of CIMP CRC. In MSI CRCs, *BRAF^V600E^* mutation is invariably associated with *hMLH1* methylation [57], and its presence, like increasing levels of CIMP, might worsen the prognosis of MSI CRC [54]. Under this respect, it should be noted that the prognostic burden of CIMP is difficult to interpret separately from the context of genomic instability in which it occurs. The tight association of CIMP with sporadic MSI CRC requires the contemporary assessment of both parameters. However, besides the potential negative effect of high level methylation on MSI CRC, MSI itself seems reverse the negative prognostic impact clearly associated with CIMP in MSS CRC [58]. To simplify, the negative impact of CIMP becomes easily evident in MSS CRC with *BRAF* mutations [53,59]. At present, the relative contributions of CIN, MSI and CIMP to the outcome of patients with CRC need to be further investigated to better understand the effects and interactions of each variable.

Hypomethylation, defined as a decrease in global DNA methylation, is also involved in colorectal carcinogenesis [60] and was associated with CIN [61,62] representing the other side of the coin of the methylation spectrum. Hypomethylation was associated with worse prognosis in one study [61], but the interplay of various types of genomic and epigenomic instability is not clear. The consequences of each type of instability at the gene level remain poorly understood, but it is reasonable to use CIN and MSI status in clinical trials to stratify patients. CIMP and global hypomethylation should be evaluated further in series stratified according to microsatellite-status, and simple markers, such as *BRAF*, which identify MSI and MSS CRC with high-level of methylation, the MSS ones clearly having a poor outcome [53,59].

## 3. Tumor Suppressor Genes and *loci*

### 3.1. Chromosome 18q, Allelic Imbalance

Allelic Imbalance (AI) or LOH is involved in colorectal carcinogenesis and occurs in the vast majority of CIN CRC (up to 85%). The LOH of chromosome 18q, an early event occurring during the adenoma-carcinoma sequence, leads to the loss of a tumor suppressor gene called *Deleted in Colon Cancer* (*DCC*) [63]. AI at 18q has been associated with a poor prognosis of stage II and III CRC patients in several studies [64-67], but these data were not confirmed by others [68,69]. Carethers *et al.* [70] published a study showing that LOH of 18q did not modify the outcome for stage II patients. The authors hypothesized that the difference in their results as compared to Jen *et al.* [64] may be due to the specific markers used in the analysis. In fact, Carethers used dinucleotides closer to and within DCC, while the microsatellite markers used by Jen *et al.* were located further away from DCC gene. In one study, Watanabe *et al.*, reported that patients with stage III MSS CRC without 18q AI had a better outcome following 5-FU based adjuvant therapy than patients with 18q AI [71]. These data suggest that the AI or LOH at 18q confers a more aggressive behavior to CRC, but the lack of validated microsatellite markers and different method used to identify 18q LOH makes it difficult to apply 18q as prognostic and predictive marker in the clinical setting. Moreover, in 18q lie other tumor suppressors involved in the process of carcinogenesis, such as *bcl*-2, *DPC4*, and *SMAD4*. Consequently, it may result difficult to precisely assess what gene damage really affects the outcome of CRC patients who carry 18q AI/LOH [72,73].

### 3.2. p53

*p53*, a tumor-suppressor gene located on the short arm of chromosome 17, is the most frequently altered gene in solid tumors. *p53* induces apoptosis or programmed cell death when damage to DNA occurs. When *p53* is altered by LOH or mutation, apoptosis does not occur and this leads to unregulated cell growth and accumulation of mutated cells. A large number of studies described the effects of genetic *p53* alterations on progression and outcome of CRC, and the results are somehow heterogeneous and conflicting, principally because of the different methodologies used to detect *p53* alterations. While Immunohistochemistry (IHC) can detect the abnormal accumulation of p53 in tumors, it does not provide clear-cut information regarding the functional status of the gene. In fact genetic analysis using direct DNA sequencing and single strand conformational polymorphisms (SSCP) showed a concordance rate with the IHC in the range of 60–85% [74]. Additional factors may account for result inconsistencies, such as small patient cohorts, IHC scoring, and investigation of non-consecutive series of patients treated with different regimens of adjuvant therapies [75].

Of the many papers showing an association between p53 alterations and worse outcome [76], 70% employed IHC and the remaining DNA analysis. Largely, it is the wide availability of antibodies for p53 IHC that has led to their use in the majority of studies assessing the relationship between p53 and survival [77,78]. The role of p53 staining as a prognostic tool in CRC has been clouded by a similar amount of studies reporting poor survival in p53-positive cases and supporting the lack of this association. Such a discrepancy exists both in studies focusing on a specific disease stage and in those including all stages. Although the sample size of the majority of studies was 100–200 patients only, the presence of several larger studies has not clarified the clinical relevance of p53 IHC. This divergence is clarified by the comparison of two papers on the utility of p53 as a prognostic marker. The first, a systematic review [79], concluded that abnormal p53 was associated with an increased relative risk of death and the effect was greatest in patients that were felt to have good prognosis. The results were similar regardless of whether ICH or DNA analyses were used. The second, a large-scale study from the International Collaborative Study of p53 and CRC [80], found that inactivating *p53* mutations were more commonly seen in more advanced tumors with lymphovascular invasion, but adversely affecting only Dukes' D tumors and not other stages. The heterogeneity of results on the prognostic value of *p53* alterations does not support its clinical use.

## 4. Oncogenes

### 4.1. KRAS

*KRAS* is a proto-oncogene that encodes 21 kDa membrane proteins with GTPase activity. It is activated in response to extracellular stimuli, and controls cellular proliferation. It is one of the earliest mutations detectable in the adenoma-carcinoma pathway in CRC development, but it can also occur at any tumor stage. It has already been detected in aberrant crypt foci [81], which are considered the first identifiable precursor lesion of CRC. Mutations in the *KRAS* oncogene have been detected in 30–50% of human CRC, 90% of the mutations occurring either in codon 12 or 13 [82]. This gene has been extensively studied in an attempt to correlate the presence and the type of a mutation with survival and response to adjuvant chemotherapy. To date, despite the large array of studies, there is no consensus considering *KRAS* as a prognostic marker. The first multicenter study assessing the implications of *KRAS* mutations on prognosis of CRC recruited 2721 patients [83]. The authors found 38% of these patients had KRAS mutations in codon 12 or 13 (90% of mutations were found in codon 12). There was no association between *KRAS* mutations and tumor location or stage, patients' geographic origin, and recurrence of disease. Interestingly, the authors showed that only the mutation glycine to valine at codon 12 adversely affects disease-free survival of CRC patients carrying this mutation. The authors confirmed this result in 2001 on a larger cohort of patients (4268 patients) [84], concluding that the presence of codon 12 glycine to valine mutation predicts a more aggressive behavior in CRC patients. Studies including a smaller number of patients reported that *KRAS* mutation was associated with worse prognosis in stage II [85] and stage III CRC patients [86]. However, recent studies reported that *KRAS* mutations have no impact on the overall survival of patients undergoing 5-FU based adjuvant therapy [87,88], or with stage IV CRC receiving the best supportive care [89]. Conclusively, the role of *KRAS* mutation as a predictive marker of response to standard chemotherapy is refuted by several studies between the *KRAS* status and the response rate to standard chemotherapy [90-93].

Differently, the predictive role of *KRAS* mutations in foreseeing response to treatment with anti-EGFR drugs has aroused great interest. A study in 30 patients with metastatic CRC treated with cetuximab demonstrated that *KRAS* mutation was found in 68% of non-responding patients but in none of the responders [94]. This is due to the fact that *KRAS* is downstream in the EGFR signaling pathway. Thus, *KRAS* mutations activate the EGFR signaling pathway independent of receptor status, bypassing the efficacy of anti-EGFR therapy. Single-arm studies [95,96] and large randomized studies on first-line [97,98] and previously treated [89,99] metastatic CRC patients have demonstrated *KRAS* tumor mutations to be predictive of a lack of response to the EGFR-targeted antibodies cetuximab and panitumumab.

*KRAS* appears to recapitulate the needs of an ideal predictive biomarker: mutations are limited to a hot spot, are easily detected, their negative predictive value is high (99% of patients with mutated *KRAS* do not respond to EGFR inhibition [89]) and the effects of the mutations are based on a plausible biological rationale.

### 4.2. BRAF

The most frequent *BRAF* mutation observed is a missense mutation leading to a valine to glutamic acid amino acid substitution (V600E). *BRAF* and *KRAS* mutations tend to be mutually exclusive events in tumors [100], with *BRAF* mutations occurring more frequently in MSI (sporadic) than in MSS tumors [51,59]. *BRAF* is functionally the most important mutation involved in the receptor-independent aberrant activation of the MEK-ERK pathway and CRC carcinogenesis. Mutations *of BRAF*, also affecting the EGFR signal transduction pathway, are found in CRC with a relatively low frequency (≤10%). As above mentioned, *BRAF^V600E^* is also tightly associated with the CIMP penotype, and the mutation can pick up almost 70% of CIMP CRC [52]. *BRAF^V600E^ has* been recently studied to clarify its role also in predicting response to anti- EGFR drugs. A study in 113 patients with wild-type *KRAS* CRC reported that patients with *BRAF*-mutated CRC had a significantly shorter progression-free and overall survival compared with those harboring wild-type gene [101]. *BRAF* mutations have been reported to be associated with poor prognosis in patients with stage IV CRC. In patients with metastatic and chemotherapy-refractory CRC, *BRAF* mutations appear to be predictive of a lack of response to EGFR-targeted agents [102]. In stage II and stage III CRC patients, *BRAF* mutations had a low frequency (7.9% of tumors) and were associated with a worse overall survival, particularly in patients with MSS tumors [88]. Thus it appears that retrospective studies highlight the fact that the association between *BRAF* mutation and poor prognosis encompasses all stages [51]. Interestingly, the better prognosis associated with MSI phenotype was mitigated by a coincident *BRAF* mutation [51,53].

In the adjuvant setting, *BRAF* mutation status appears to be a valid predictive marker. However, associations of *BRAF* mutations with different molecular subgroups of CRC should be considered in order to assess the impact of *BRAF* status as a predictive marker for treatment in future studies.

## 5. Candidate Biomarkers in Colon Cancer

### Cancer Microenvironment: Prognostic Information of Immune Cell Infiltrate

As our understanding expands, it is becoming clear that neoplastic clones and their molecular alterations cannot entirely explain cancer behavior. The other emerging player is the tumor microenvironment with its load of fibroblasts, endothelial and immune cells, and related cytokines, all of which have an important role in the development and progression of solid tumors. The immune system acts in a protective way against the development of tumors, exerting an active immunosurveillance [103]. Additional evidence supports, both in humans and animals, the role of immune cells in controlling late progression and metastasis [104,105]. The prognostic impact of immune cells in CRC, perceived almost 30 years ago [106], was re-addressed by Naito *et al.* [107], demonstrating that a high density of intraepithelial CD8+ cells within the tumor correlated with an improved survival. When the density of the infiltrating nests were scored, a high density of CD8+ cells independently provided equivalent prognostic ability as TNM staging. Subsequently, the abundance of tumor infiltrating T-cells has been associated with improved CRC patient outcome in several papers [108-129]. Collectively, it has been suggested that prognosis in patients with cancer is positively affected by (a) the presence of a tumor gene signature consistent with a type I adaptive immune response *(i.e.*, increased antigen presentation, IFN-γ signaling, and T cell receptor signaling), and (b) the presence of T cells that penetrate through tumor stroma and deeply infiltrate the parenchyma to become intra-tumoral T cells. Thus, besides a Th-1 response signature, the other key feature of an effective immune response is the ability of T cells to travel to the site of the tumor and infiltrate it. Because T-cell infiltration is not homogeneous in CRC, attention has been focused on the predictive value of T cells in the *center of the tumor*, in the *invasive margin* and in lymphoid islets located at the vicinity of the tumor (*tertiary lymphoid structures*, complete with both T cells and DCs).

Two large studies [115,121] assessed the immune component of the tumor microenvironment by a combination of high-throughput gene expression and immunophenotypic analyses and evaluated its possible influence on tumor dissemination. The authors found that their data support the hypothesis that adaptive immune response influences the behavior of human tumors. *In situ* analysis of tumor-infiltrating immune cells may therefore be a valuable prognostic tool which might even be superior to TNM staging, according to specific studies [115,130]. Independently, Deschoolmeester *et al.* [111] showed that the presence of a pronounced lymphocytic infiltration within the tumor is associated with improved survival. They found that CD3^+^ and CD8^+^ T lymphocytes within tumor glands, and of CD3^+^ in the stroma, had a major impact on the patients' overall survival. The improved survival associated with infiltration of T lymphocytes has been suggested to result from the effective suppression of micrometastases. Therefore, the densities of CD8^+^ T cells within the primary tumor might be a good indicator of the presence of a systemic immunesurveillance mechanism. Previous studies have reported that MSI, CIMP, *BRAF* mutation, *PIK3CA* mutation, and *LINE-1* tumor hypomethylation were associated with CRC prognosis, and that lymphocytic infiltration is associated with many of these molecular variables [61,131,132]. The association of a prognostic biomarker with a given disease stage strongly suggests its stage-dependency as outcome predictor. This is best exemplified by MSI CRC, whose overall prognostic advantage is associated with a low frequency of stage III and IV cases [39] at diagnosis as compared to the microsatellite stable counterpart. Most MSI CRCs show a pronounced intratumoral inflammatory reaction [116,117,119,124,125,133,134], the mechanistic explanation of which, however, is still incompletely understood. Infiltrating lymphocytes have been identified within these tumors as being predominantly activated CD8^+^ T cells. The presence of these cytotoxic T lymphocytes has been attributed to the inherently greater production of abnormal peptides as a result of unreliable DNA repair in MSI-positive tumors [135]. Truncated peptides produced by frameshift mutations due to MSI may be immunogenic and contribute to the host immune response. However, little is known about the interrelationship between tumor-infiltrating T cells, MSI, and other molecular features of CRC, including CIMP, global DNA hypomethylation, or *KRAS*, *BRAF* and *PIK3CA* mutations. It is indubitable that to define the prognostic effect of tumor-infiltrating T-cells independently of other potential confounders, more large studies incorporating an extensive molecular characterization of CRC are needed. Additionally, caution is required, before incorporating tumor-infiltrating T cells into tumor staging, to minimize the risk of inappropriate tumor down-staging at diagnosis, and survival data need to be confirmed in independent series of patients collected in the last decade. Moreover, the association has to be conclusively proven between low densities of tumor-infiltrating T cells and the clinical detection of metachronous metastases, which remains the most appropriate outcome measure for recognizing the role of local immune response in micrometastasis suppression. Recently, Laghi *et al.* [117] investigated the relationship between the density of CD3^+^ T infiltrating lymphocytes at the invasive margin of the tumor, and the occurrence of metachronous distant-organ metastases after potentially curative resection, in a large, consecutive series of patients with deeply invading (pT3 or pT4) MSI-typed CRC, and no evidence of distant organ metastasis at diagnosis. They found that large areas of CD3^+^ cells at the invasive front of pT3 or pT4 CRCs are associated with low risk of metachronous metastasis and consequently a survival advantage only in patients with node-negative cancers, but not in patients whose cancers involved lymphnodes. Of interest, the prognostic advantage conferred by high density CD3^+^ cells was independent of tumor MS-status in patients with stage II CRC. CD3-immunostaining of CRC tissue might therefore be useful in selecting stage II patients who, because they are at very low risk for cancer progression, could be spared adjuvant treatments. Conflicting data exist on what concerns tumor-infiltrating FOXP3^+^ Tregs. Nosho *et al.* [136] examined the prognostic role of tumor-infiltrating T-cell subsets (CD3^+^, CD8^+^, CD45RO^+^, and FOXP3^+^) in a large series of 768 CRCs from two prospective cohort studies. They found that the density of CD45RO^+^ cells, but not that of CD3^+^, CD8^+^, or FOXP3^+^ cells, was an independent prognostic biomarker of longer survival in CRC patients. In contrast, Salama *et al.* [125] by analyzing T-cell infiltrates in 967 CRCs including 593 stage II and 374 stage III cases, reported that FOXP3^+^ Treg density had stronger prognostic significance than CD8^+^ and CD45RO^+^ lymphocytes, and predicted a better outcome. FOXP3^+^ Tregs were found not associated with any histopathologic features except tumor stage. At multivariate analysis, stage, vascular invasion, and FOXP3^+^ Treg density in tumoral tissue were independent prognostic indicators. These results led the authors to conclude that inclusion of FOXP3^+^ Treg density may help to improve the prognostication of early-stage CRC. Even in this study, they did not explore further the stage independence of the immune population, which curiously can originate from either the tumor centre or its invasive margins.

It is undisputed in this field that the adaptive immune infiltrate exerts a protective role on cancer progression, likely restraining the development of metastasis. However, besides still conflicting data, the lack of a standard methodology (e.g., tissue microarray *vs.* whole section analysis, image acquisition and assessment), coupled with the study of different regions (centre *vs.* margin of the tumor), and with overlapping of T lymphocyte subpopulations loaded with prognostic impact, call for a critical reappraisal of current data and specific improvements.

Data concerning the influence of individual's acquired antitumor immunity has on the development and progression of CRC, are compelling and demonstrate a clear interaction between neoplastic growth and patient response to deal with this threat. Unfortunately, given that a better understanding of the tumor immunity response is needed, methodological issues need to be settled before these parameters can enter the clinical arena.

## 6. Future Perspectives

### Epithelial to Mesenchymal Transition (EMT): A New Potential Source of Prognostic Markers

Development of distant metastases, the final stage of solid cancer progression, is responsible for the majority of cancer-related deaths [137]. While clinically of greater importance, the biology of metastasis remains unsolved. The process of tumor metastasis consists of multiple steps, all of which are required to achieve tumor spreading [138,139]. Firstly, cancer cells leave their original site by breaking the basal membrane. These cancer cells then invade the tumor stroma and enter the blood circulation, either via the lymphatic system or by intravasation. Most circulating cancer cells undergo apoptosis due to anoikis (Anoikis is a form of programmed cell death induced in anchorage-dependent cells by the detachment from the surrounding extracellular matrix) conditions [140]. If cancer cells survive in the circulation they may reach more suitable sites by attaching to endothelial cells and extravasating from the circulation into the surrounding tissue. Finally, distal colonization requires that cancer cells invade and grow in a new environment. Recently, the concept of the epithelial-mesenchymal transition (EMT), initially developed in the field of embryology, has been extended to cancer progression and metastasis [141,142], as changes in cell adhesion and migration of tumor invasion are reminiscent of crucial developmental processes. Epithelial cancer cells are thought to acquire a more migratory mesenchymal-like phenotype in order to undergo intravasation and to complete all the stages of the metastatic process. During EMT, epithelial markers are lost in favor of mesenchymal ones, and the morphology of the epithelial cell changes. Data from *in vitro* and animal models support the hypothesis that EMT plays a role in the metastasis process. Several studies have clarified the biology of EMT in tumor samples by studying EMT-associated markers, such as the appearance of mesenchymal ones (vimentin and fibronectin) [143,144], coupled with the loss of epithelial markers (E-cadherin and cytokeratin) [145,146]. Additionally, various transcription factors (Snail and Slug) are switched on [147]. Multiple complex signaling systems are required for the induction of EMT, to drive the cells through morphological changes. One crucial change is the regulation of E-cadherin. Cells in EMT show a decreased expression of E-cadherin, and it is widely accepted that the loss of this ephithelial marker can lead to tumor progression and metastasis in various human carcinomas [145,146,148,149]. Genetic and epigenetic alterations cause a functional loss of E-cadherin [150-153]. Interestingly, Graff *et al.* [152] showed that the degree of methylation of the E-cadherin promoter can be unstable and heterogeneous during the progression of metastasis, suggesting that E-cadherin loss, in primary cancer, may lead to metastasis by EMT activation. EMT can be induced also by TGF-β [154]. Several data have shown that TGF-β induces multiple responses in cancer progression [155]. Basically, TGF-β is a strong growth inhibitor [156], and its loss can result in cancer progression. Indeed, Hahn *et al.* reported that mutations in *TGF-b* and *Smad4* can give rise to pancreatic [73] and CRC [157]. In addition, TGF-β can induce EMT through multiple signaling pathways, including the phosphorilation of Smad2 and Smad3. It should be noted that inability to exploit EMT, largely ascribed to the lack of functioning TGFβ-RII, can help to explain the low metastatic potential of MSI CRC [158].

The tumor micro-environment is composed of extracellular matrix (ECM) fibroblasts, myofibroblasts, immune cells, and soluble factors, all of which can act on cancer progression. The interactions between cancer cells and microenvironment can induce EMT by auto and/or paracrine secretion of mediators such as growth factors, cytokines and ECM proteins [159]. In a comparison of the central areas of primary CRC and their matched metastases, nuclear β-catenin localized to the membrane and cytoplasm, was detected in dedifferentiated mesenchymal-like tumor cells at the invasive front [160]. This study suggests that the tumor microenvironment may induce, maintain or otherwise cooperate in EMT.

Transcriptional repressors of E-cadherin such as zinc finger proteins (ZEB1, ZEB2), and the snail family of zinc finger proteins (Snail and Slug) are associated with EMT [161-164]. Several transcriptional factors such as Snail, Slug and recently Twist1 [165], are moving from basics to translational studies as candidate prognostic markers in various human carcinomas.

Twist1 is a highly conserved transcription factor that belongs to the family of basic helix-loop-helix (bHLH) proteins. In vertebrates, it is involved in embryonic development through the regulation of epithelial–mesenchymal transitions (EMT) during neural crest migration, and regulates mesoderm determination, myogenesis, and morphogenesis [166,167]. It has been suggested that Twist1 is oncogenic, contributing to metastasis through its involvement in EMT regulation [165]. Inhibition of Twist1 in metastatic mammary carcinoma cells suppresses their ability to migrate from the mammary gland to the lung, in a mouse model. Ectopic expression of TWIST1 results in the loss of E-cadherin-mediated cell to cell adhesion, activation of mesenchymal markers, and induction of cell motility [165]. Twist1 is overexpressed in multiple tumor types, and it is associated with poor prognosis. Elevated levels of Twist1 mRNA have been observed in rhabdomyosarcomas [168], diffuse-type gastric carcinomas [169], breast cancer [165], neuroblastomas with N-myc amplification [170], gliomas [171], oesophageal squamous cell carcinoma [172], and pancreatic cancer [173]. IHC analyses showed that tumors express Twist1 at elevated levels compared with normal tissue in several human malignancies [174-180]. All this evidence suggests that Twist1 is a common oncogene playing a key role in cancer invasion.

Twist1 expression has not yet been characterized in human primary colorectal tumors, but recently Valdes-Mora *et al.* [181] reported upregulation of *Twist1* mRNA in CRC, suggesting its implication in disease progression. In addition, they reported that significant higher levels of *Twist1* mRNA were found in men than in women, suggesting a possible transcriptional regulation of *Twist1* by sexual hormones. They also proposed the use of *Twist1* as a new prognostic marker of advanced malignancy, and as a potential therapeutic target in CRC.

EMT-associated markers in clinical samples have been identified in histological specimens. However, the existence of EMT cells in clinical specimens has been challenged [182]. In response, Voulgari *et al.* suggested that the controversy between experimental and clinical studies might be due to the ‘spatial’ and ‘temporal’ heterogeneity of EMT [183]. Cells undergoing EMT may gain metastatic potential but may constitute only a small proportion of the total population of cancer cells, and could have characteristics of cancer stem cells (CSC) [184]. Therefore, it is difficult for the pathologist to identify those cancer cells undergoing EMT in clinical specimens. The temporal heterogeneity of EMT (and its potential reversibility) is readily explained. EMT reversibility is observed *in vitro* following the addition of bone morphogenetic protein 7 (BMP7), removal of an EMT-inducer such as TGF-β, and establishment of hypoxic conditions [185,186]. A similar process may occur at metastatic sites which require cancer cells to recover the expression of E-cadherin for cell adhesion. Their reversibility could make it difficult to prove that EMT, a transient phenomenon involving only a minority of cells, has occurred in human cancer specimens. However, EMT-associated genes are obviously worth being tested as biomarkers. Clinical verification of EMT will require advanced techniques such as *in vivo* imaging, and is expected to shortly provide new insights into prognostic and predictive biomarkers.

## 7. Conclusions

The improvement in patient outcome observed over the past 20 years has been accompanied by a large number of candidate prognostic and predictive markers, the vast majority of which failed to demonstrate clinical utility. Many more molecular markers of prognosis than those discussed in this review have already been described. Clearly a better validation is needed before any of these markers can be associated with prognosis or with the response to therapy, and therefore incorporated into clinical practice. Too often inconsistencies arise between initial reports and subsequent studies, due to different assays, study design and/or statistical methodologies [15]. The prognostic effect of the molecular phenotype, initially described many years ago, has only recently entered into prospective clinical trials. Currently, the only markers with sufficient evidence to justify routine clinical assessment are *KRAS* selection for EGFR-specific therapy, plus MSI. The latter can be considered a prognostic marker which in appropriate clinical settings can be helpful in clinical decision making, although its effectiveness is limited by the stage dependency [39]. It is clear that a much greater degree of cooperation is required between basic and clinical scientists, to bring sufficient rigor to this field and to the designing of trials with enough statistical power to provide results that will compel clinical engagement.
